# Acceleration of atherogenesis in *ApoE^−/−^* mice exposed to acute or low-dose-rate ionizing radiation

**DOI:** 10.18632/oncotarget.5075

**Published:** 2015-08-22

**Authors:** Mariateresa Mancuso, Emanuela Pasquali, Ignacia Braga-Tanaka, Satoshi Tanaka, Alessandro Pannicelli, Paola Giardullo, Simonetta Pazzaglia, Soile Tapio, Michael J. Atkinson, Anna Saran

**Affiliations:** ^1^ Laboratory of Radiation Biology and Biomedicine, Agenzia Nazionale per le Nuove Tecnologie, l'Energia e lo Sviluppo Economico Sostenibile (ENEA), Rome, Italy; ^2^ Institute for Environmental Sciences (IES), Rokkasho, Aomori, Japan; ^3^ Technical Unit of Energetic Efficiency, ENEA, Rome, Italy; ^4^ Department of Radiation Physics, Guglielmo Marconi University, Rome, Italy; ^5^ Department of Sciences, Roma Tre University, Rome, Italy; ^6^ Helmholtz Zentrum München, German Research Center for Environmental Health, Institute of Radiation Biology, Neuherberg, Germany

**Keywords:** atherosclerosis, ApoE mice, radiation, aorta

## Abstract

There is epidemiological evidence for increased non-cancer mortality, primarily due to circulatory diseases after radiation exposure above 0.5 Sv. We evaluated the effects of chronic low-dose rate *versus* acute exposures in a murine model of spontaneous atherogenesis. Female *ApoE−/−* mice (60 days) were chronically irradiated for 300 days with gamma rays at two different dose rates (1 mGy/day; 20 mGy/day), with total accumulated doses of 0.3 or 6 Gy. For comparison, age-matched *ApoE−/−* females were acutely exposed to the same doses and sacrificed 300 days post-irradiation. Mice acutely exposed to 0.3 or 6 Gy showed increased atherogenesis compared to age-matched controls, and this effect was persistent. When the same doses were delivered at low dose rate over 300 days, we again observed a significant impact on global development of atherosclerosis, although at 0.3 Gy effects were limited to the descending thoracic aorta. Our data suggest that a moderate dose of 0.3 Gy can have persistent detrimental effects on the cardiovascular system, and that a high dose of 6 Gy poses high risks at both high and low dose rates. Our results were clearly nonlinear with dose, suggesting that lower doses may be more damaging than predicted by a linear dose response.

## INTRODUCTION

Radiation is a risk factor in human vascular disease and studies on atomic bomb survivors cohort indicate that non-cancer disease mortality, particularly cardiovascular disease, contributes to the same degree as cancer mortality to the radiogenic excess risk [1]. An increased incidence of atherogenesis is also observed after radiotherapy in survivors of Hodgkin's disease, breast cancer, and head and neck cancer [2]. The ICRP 2011 raised attention to circulatory disease at low doses [3] because of recent evidence of increased risks not only at doses above 5 Gy but also in a range of doses from 5 to 0.5 Gy and possibly even at lower doses (<0.5 Gy) which might be exceeded during complex radiological investigations [4–7].

Cardiovascular effects due to low dose-rate radiation cannot be accurately predicted from responses to acute high dose-rate exposures [8, 9] since the biological responses (e.g., cellular repair) relevant to cardiovascular disease may change over the protracted irradiation time.

Atherosclerosis is an inflammatory disease of the arteries that can lead to ischemia of the heart and brain, resulting in infarction [10]. The apolipoprotein E-deficient (*ApoE^−/−^*) mice exhibit marked increases in plasma cholesterol levels when fed a normal chow diet and are predisposed to spontaneous atherogenesis, with lesions resembling those seen in humans. Therefore, they represent a useful animal model for studying the role of radiation in the atherogenic process *in vivo* [11, 12].

Using the low dose-rate facility at the Institute of Environmental Sciences (IES), Rokkasho, Japan, we have conducted a series of long-term *in vivo* studies investigating the effects of different chronic exposure rates on the cardiovascular system. These effects were compared with those due to acute exposure at the same total dose.

## RESULTS

### Acute irradiation

At 6 Gy, we observed acute mortality in 25% (6/24) mice within 10 days from irradiation. A total of 18 mice survived and were assigned to different experimental groups (see Methods and Materials).

**Figure 1:**
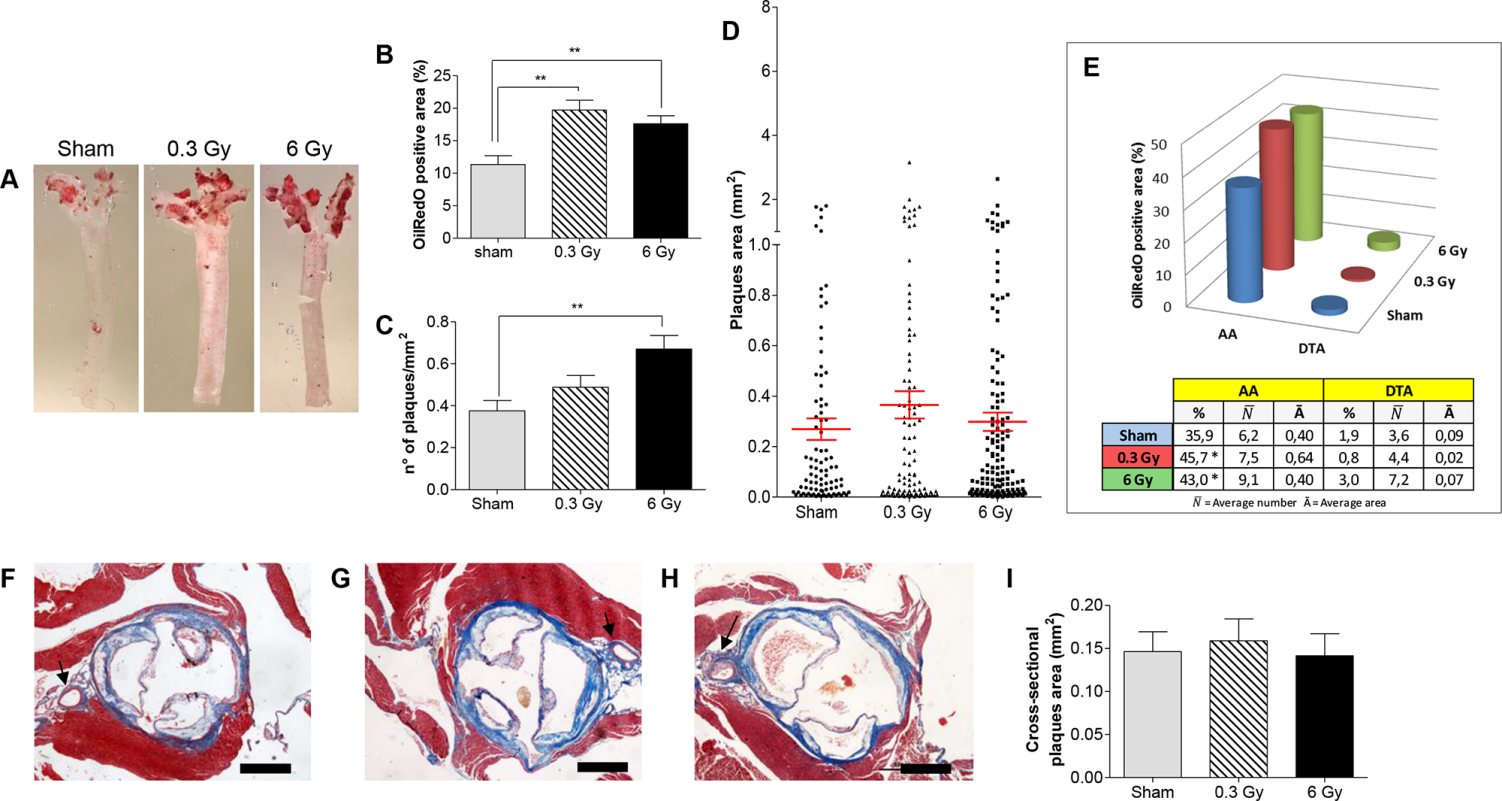
Acute irradiation **A.** Representative *en face* preparations of aortas from female *ApoE^−/−^* mice 300 days after acute irradiation with 0.3 Gy, 6 Gy and from age-matched controls. **B–D.** Morphometric analyses performed on digital images from *en face* preparations of aortas (*n* = 8) representing percentage of ORO-stained area (B), plaques density (C) and plaques size (D). **E.** Regional distribution of plaques. **F–H.** Representative aortic root sections from sham- (F), 0.3 Gy- (G) and 6 Gy-irradiated mice (H) (Masson's trichrome stain). **I.** Graphic representation of plaques area (*n* = 18) measured on aortic root cross-sections. Data are shown as mean ± SEM. Differences were tested with Student's *t*-test. **P < 0.001. Arrows: coronary artery. Bars: 500 μm.

For quantitative analysis of plaque area and number, we carried out dimensional analyses of digital images from *en face* ORO-stained thoracic aortas (*n* = 8) (Fig. 1A). The mean percentage of plaque-covered aorta area was significantly increased at 300 days from acute exposure to both x-ray doses (*i.e*., 0.3 and 6 Gy), compared with sham-irradiated mice (Fig. 1B; *P* < 0.05). Two different factors, number of plaques and their size, can contribute alone or in combination to the increased amount of lipid-laden area in irradiated *ApoE^−/−^* mice. Fig. 1C shows that irradiation at all doses caused a general increase in density of atherosclerotic lesions (number of plaques/mm^2^), although in mice irradiated with 0.3 Gy this was not statistically significant (1.3-fold; *P* = 0.1501). In the same group, there was a 1.4-fold increase in plaque size compared with controls (Fig. 1D; 0.36 ± 0.054 *versus* 0.26 ± 0.042; *P* = 0.1761). Although this difference was again not significant, the combination of both factors (number/area of plaques) accounts for the statistical increase in terms of ORO-stained total area detected at the lower 0.3 Gy dose. At 6 Gy the density of atherosclerotic lesions increased significantly (1.8-fold; *P* = 0.0022), while plaque size remained very similar to that measured in sham-irradiated mice (Fig. 1D; 0.29 ± 0.036 *versus* 0.26 ± 0.042).

Atherosclerotic plaques developed principally in the aortic arch (AA), a region presenting both a high curvature zone and bifurcations [13]. Fig. 1E shows the regional distribution of ORO-stained plaques in sham-irradiated *ApoE^−/−^* mice, with lesions located predominantly in the AA region (35.9% $\bar{N}=6.2$ $\bar{A}=0.40\pm 0.06$) and rare plaques in the descending thoracic aorta (DTA) (1.9% $\bar{N}=3.6$ $\bar{A}=0.09\pm 0.02$). A similar regional distribution was observed after irradiation. However, the lipid-laden area in the AA increased with irradiation. In fact, mice exposed to 0.3 Gy showed a 1.6-fold increase in plaque area in the AA (45.7% $\bar{N}=7.5$ $\bar{A}=0.64\pm 0.07$) although there was no variation in plaque number. In contrast, mice irradiated with 6 Gy showed a 1.5-fold increase in number of AA plaques and no size variation (43.0% $\bar{N}=9.11$ $\bar{A}=0.40\pm 0.05$). Mice irradiated with 6 Gy showed presence of plaques in the DTA region (3.0% $\bar{N}=7.2$; $\bar{A}=0.07\pm 0.01$), although the increase in number was not significant. Altogether, these results demonstrate that an acute dose of radiation increases atherogenesis in *ApoE^−/−^* mice, suggesting that the mechanisms for plaque formation is different between low and high doses of radiation, acting as plaque promoting or initiating stimulus, respectively.

Cross-sectional analysis of the aortic root allows measurement of endpoints relevant for processes involved in plaque progression (*i.e*., stenosis and plaque vulnerability). First, we quantified the average lesion size in cross-sections through the aortic root (*n* = 18 per group; Fig. 1F, 1G and 1H). As shown in Fig. 1I, we did not observe any significant increase in average lesion area in irradiated mice compared to controls. In the coronary artery, plaque formation was observed only after acute irradiation with 6 Gy.

**Figure 2:**
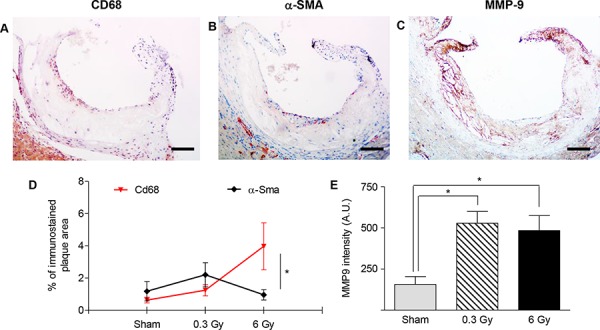
Plaques vulnerability after acute irradiation **A–C.** Representative sections of atherosclerotic plaques immunostained with antibodies against CD68 (A), α-SMA (B) and MMP-9 (C). Images refer to *ApoE^−/−^* mice 300 days after acute irradiation with 6 Gy. **D.** Mean percentage of total plaque area occupied by CD68- or α-SMA-positive cells. **E.** Intensity measurement of anti-MMP9 immunohistochemical staining by HistoQUEST software. Quantitative analysis involved 18 plaques/group. Data are shown as mean ± SEM. Differences were tested with Student's *t*-test. *P < 0.05. Bars: 100 μm.

We then quantified the amount of macrophages, smooth muscle cells (SMCs) and macrophage-derived matrix metalloproteinase-9 (MMP-9) in serial sections of lesions through the aortic root (Fig. 2A, 2B and 2C). Fig. 2D shows the mean percentage of plaque area occupied by macrophages (CD68-positive cells) and SMCs (α-SMA-positive cells). In mice irradiated with 6 Gy, plaques showed typical features of more advanced lesions as indicated by a high percentage of CD68-positive cells *versus* fibrous-cap thinning (low percentage of SMCs) (*P* = 0.0497). Conversely, in sham- and 0.3-Gy-irradiated groups these parameters were very similar. However, irradiation at both doses significantly increased the expression of MMP-9, a protease-activated enzyme involved in degradation of the extracellular matrix.

### Chronic irradiation

The spontaneous level of atherosclerotic lesions, as evaluated by ORO stain in 360-days old *ApoE^−/−^* mice maintained at the IES animal facility, was 4-fold higher than that observed in the age-matched *ApoE^−/−^* mice housed at ENEA (44.8% *versus* 11.3%; *P* < 0.0001). The baseline regional distribution of plaques was also very different. Intriguingly, mice were purchased from the same producer and belonged to the same batch (shipped to the two laboratories at the same time), and fed the same diet, therefore we hypothesize that different housing conditions (SPF *versus* conventional) strongly influenced atherosclerosis [14]. In addition, other factors in standard laboratory practice may represent a confounding factor when investigating phenomena possibly affected by stress [15]. Despite baseline differences, it is worthwhile to compare atherogenic radiation responses in the two colonies.

**Figure 3:**
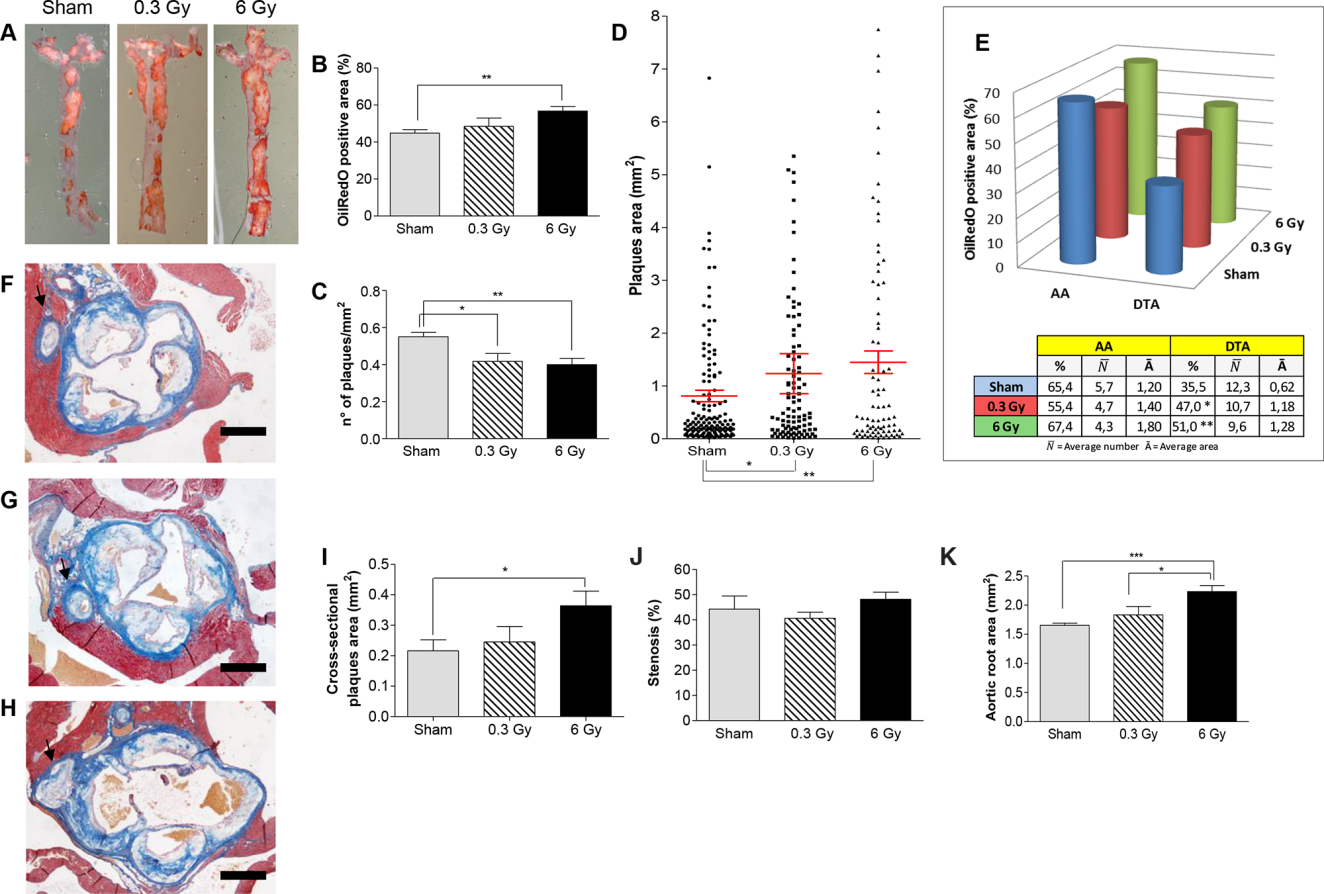
Chronic irradiation **A.** Representative ORO-stained aortas from female *ApoE^−/−^* mice after chronic irradiation with 0.3 Gy or 6 Gy over 300 days, and age-matched controls. Graphic representation of quantitative analyses performed on digital images from en face preparations of aortas (*n* = 8) showing: **B.** Percentage of ORO-stained aortic area. **C.** Plaque density. **D.** Plaques size. E. Regional distribution. **F–H.** Masson's trichrome staining of aortic root cross-sections from each experimental group. **I–K.** Dimensional analyses of plaques area (I), aortic stenosis (J) and aortic total area (K). Data are shown as mean ± SEM. Differences were tested with Student's *t*-test. *P < 0.05; **P < 0.001; ***P < 0.0001. Arrows: coronary artery. Bars: 500 μm.

After chronic irradiation, the mean percentage of thoracic aorta area covered by plaque was significantly increased, compared with controls, only in mice exposed to 6 Gy (Fig. 3A and 3B; *P* = 0.0020). Although the density of plaques showed significant decrease both after 0.3 Gy (0.55 ± 0.02 *versus* 0.41 ± 0.04; *P* = 0.0318) and 6 Gy (0.39 ± 0.03; *P* = 0.0024), compared with controls (Fig. 3C), the mean plaque area was significantly higher in both irradiation groups, which for the 6-Gy dose resulted in significant overall increase. We found 1.5- (*P* = 0.0401) and 1.8-fold (*P* = 0.0034) increases in plaque area in 0.3- and 6-Gy irradiated mice, respectively, over controls (Fig. 3D), which suggests coalescence of existing or *de novo* plaques resulting in apparent decrease in frequency (Fig. 3C).

Regional distribution analysis of plaques (Fig. 3E) showed a very high percentage of AA area covered by plaques in control mice (65.4% $\bar{N}=5.75$ $\bar{A}=1.2\pm 0.25$). The DTA region also showed dramatic involvement in the atherogenic process (35.5% $\bar{N}=12.3$ $\bar{A}=0.62\pm 0.19$). Because of massive presence of plaques in the AA, the effect induced by chronic irradiation was more easily assessed as percentage of ORO-stained area in the DTA. As shown in Fig. 3E, chronic irradiation significantly increased the mean percentage of DTA covered by plaques after both 0.3 and 6 Gy (47% and 51%, respectively). This global increase was due to 1.9-fold and 2.0-fold increases in plaque size in 0.3 Gy- ($\bar{N}=10.7$ $\bar{A}=1.18\pm 0.24$ *P* = 0.0205) and 6 Gy-irradiated mice ($\bar{N}=9.6$ $\bar{A}=1.28\pm 0.24$ *P* = 0.0064), respectively, relative to controls. Moreover, the 6-Gy dose was more efficient in promoting atherogenesis, as shown by cross-dimensional analyses of the aortic root (Fig. 3F–3I). A statistically significant increase in cross-sectional area of the plaques was observed only in this group compared with age-matched controls (Fig. 3I; *P* = 0.0255). Nevertheless, we did not observe stenosis (Fig. 3J) due to a phenomenon of arterial remodeling, as indicated by significant increase of aortic root total area (Fig. 3K). Therefore, our data indicate that plaque progression is poorly correlated to luminal size, as previously observed in humans [16]. Notably, the coronary arteries were severely affected with plaques, especially in the 6 Gy group (Fig. 3F–3H).

**Figure 4:**
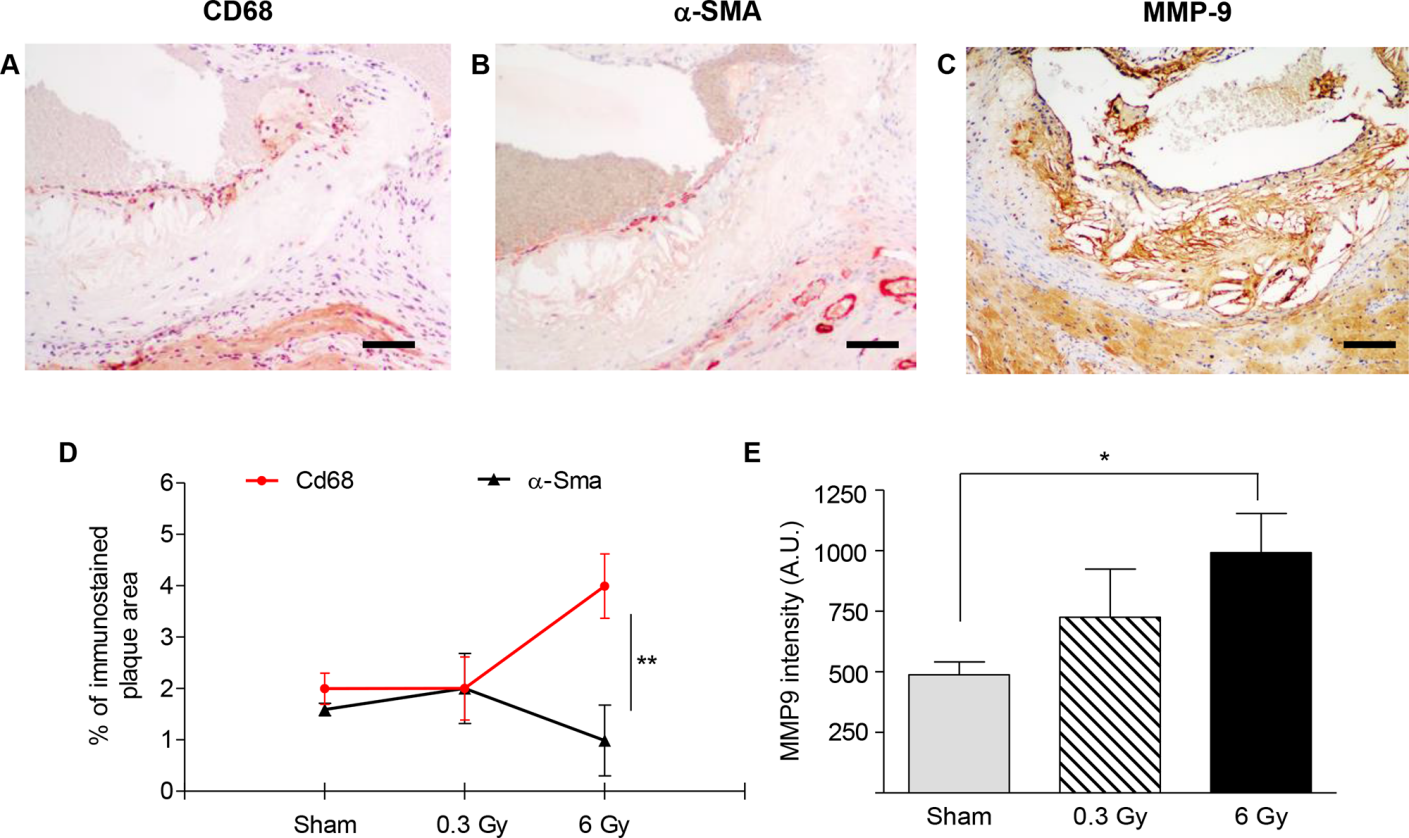
Plaques vulnerability after chronic irradiation **A–C.** Representative sections of atherosclerotic plaques after chronic irradiation with 6 Gy using antibodies anti-CD68 (A), α-SMA (B) and MMP-9 (C) **D.** Mean percentage of total plaque area occupied by CD68- or α-SMA-positive cells. **E.** Intensity measurement of immunohistochemical staining with anti-MMP9 by HistoQUEST software. Quantitative analysis involved 18 plaques/group. Data are shown as mean ± SEM. Differences were tested with Student's *t*-test. *P < 0.05; ***P < 0.0001. Bars: 100 μm.

Importantly, immunostaining of plaques with anti-CD68, anti-α-SMA and anti-MMP-9 (Fig.4A–4C) showed again more advanced lesions after 6 Gy. At this dose, the percentage of plaque area occupied by macrophages was significantly higher compared with the percentage area of SMCs (*P* = 0.0071) (Fig. 4D), indicating altered balance between inflammation and fibrosis during plaque progression. Moreover, significantly increased MMP-9 expression was found in the 6-Gy group.

## DISCUSSION

Atherosclerosis is a multifactorial disease, resulting from interactions between genetic and environmental factors, and is susceptible to modification by radiation exposure [17]. Epidemiological evidence has established links between cardiovascular disease and exposure of the heart and major vessels to doses above 0.5 Gy. At lower doses, the evidence for detrimental effects remains inconclusive [18]. Partly, this is due to the lack of appropriate epidemiological studies, and/or lack of knowledge of the biological processes involved in development of cardiovascular diseases following irradiation. Furthermore, there is limited availability of experimental data in animals.

The influence of dose rate on late health effects remains controversial, and there is no universal European or International acceptance of a dose rate correction factor. Cardiovascular effects of low doses have been seen in chronically irradiated cohorts [(e.g., Mayak workers] [8, 19] but estimates of the influence of dose rate on risks at low doses are missing.

We have conducted long-term *in vivo* studies on the effects of different chronic exposure rates (1 and 20 mGy/day) on the cardiovascular system using the IES low dose rate mouse exposure facility designed to continuously expose mice under SPF conditions to ^137^Cs gamma rays at low/very low dose rates, aiming to compare results with those due to the same cumulative doses delivered acutely.

We used *ApoE^−/−^* mice, a widely used model of human atherosclerosis, and exposed them to a high dose (6 Gy), which is relevant to cancer therapy, or to a moderate dose (0.3 Gy), such as might occur in high-dose diagnostic procedures or worst-case scenario occupational exposures (*e.g*., nuclear workers). These two doses were given acutely, or protracted over 300 days, to gain insights into the effects of low dose and/or chronic radiation exposures on cardiovascular disease, an area of major uncertainty. This is, to our knowledge, the first study of radiation induced cardiovascular disease addressing extremely low dose rates, where cell death of critical inflammatory cell populations will be minimized and cellular repair mechanisms maximized [6].

The study was designed to address very specific questions in radiation protection applied to heart disease: [1] Is there clear evidence for detrimental or protective effects on the cardiovascular system at low dose? [2] Are there protective/detrimental effects of dose rate? [3] What are the biological mechanisms underlying low-level radiation effects and are they different at high doses/dose rates?

Our results reveal that after acute exposures to both 0.3 Gy and 6 Gy there was a significant increase in plaque coverage compared with unexposed *ApoE^−/−^* mice, by *en face* analysis of aortas. Whereas at 0.3 Gy this was due to combined effects of increased plaque number and enlarged plaque size, at 6 Gy there was primarily an increased lesion frequency, which was accompanied by contribution of the DTA to total plaque coverage. Effects were very persistent, being evident 300 days after exposure. Thus, both an acute high dose of 6 Gy and a moderate 0.3 Gy dose can have a significant impact on development of atherogenesis in a predisposed mouse model, although with different mechanisms, *i.e*., predominantly lesion formation at high doses and growth of existing lesions at low doses.

Chronic irradiation also affected atherogenesis at both doses compared with unirradiated mice. In mice exposed to 0.3 Gy there was a modest increase in percentage of ORO-stained area and a significant decrease in plaque density. This, however, was due to increased plaque size and consequent coalescence, rather than genuine reduced lesion frequency. In fact, by restricting the analysis to the DTA, a significant increase of ORO-stained area was evident compared with sham-irradiated mice. At 6.0 Gy there was significant increase in both mean percentage of thoracic aorta area covered by plaque and increased plaque size. Again, the reduced plaque density appeared to be linked to the plaques increased size, making it difficult to distinguish separate lesions. In this group we also observed increased cross-sectional area of the plaques, as well as the presence of plaques in the coronary artery.

**Figure 5:**
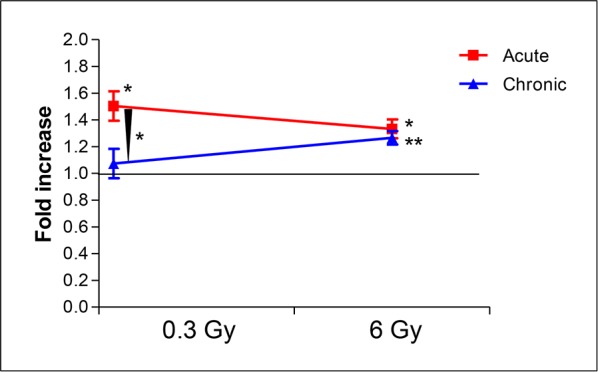
Comparison of fold increases of percent ORO-stained aortic area over sham-irradiated controls between high (x rays) and low dose-rate (gamma rays) groups Assuming a quality factor of 1 for x rays and gamma rays, a high radiation dose (6 Gy) would be equally efficient in enhancement of atherogenesis at both high and low dose rate. In contrast, a moderate dose (0.3 Gy) could significantly enhance atherogenesis only when delivered acutely (*P* = 0.0143, acute versus sham irradiated). When comparing fold increases, mice receiving 0.3 Gy acutely have enhanced atherogenesis compared with mice receiving the same dose as a chronic exposure (*P* = 0.0298).

Although direct comparisons of the extent of atherogenesis between high and low dose-rate groups cannot be made, due to differences in animal husbandry and health status of control mice at the two facilities, our results can nevertheless be interpreted correctly based on the fold increase in atherosclerosis (%ORO-stained area) in irradiated mice compared to their own sham-irradiated groups, maintained at the same facility. Direct comparisons are also hampered by the different radiation exposure deliveries. Indeed, despite the fact that a quality factor of 1 is assigned for both x rays and gamma rays, the reliability of this assigned value has been called into question [20, 21], more so for different tissues *in vivo* [21]. Within these acknowledged limitations, a tentative comparison is worth presenting, as it may help gain insights into this area of radiobiological research where much uncertainty persists. Fig. 5 shows that, given a quality factor of 1 for x and gamma rays, a high radiation dose (6 Gy) would be equally efficient in enhancement of atherogenesis at both high and low dose rate, whereas a moderate dose (0.3 Gy) could significantly enhance atherogenesis only when delivered acutely (*P* = 0.0143, acute *versus* sham irradiated). When comparing fold increases, mice receiving 0.3 Gy of x rays acutely had enhanced atherogenesis compared with mice receiving 0.3 Gy of gamma rays as a chronic exposure (*P* = 0.0298). At 6 Gy, no such effect of dose rate would be detectable.

An important aspect of our results is the more progressed status of plaques in mice irradiated with 6 Gy regardless of dose rate, shown by CD68, α-SMA and MMP-9 immunoreactivity. In particular, we showed increase in plaque vulnerability and proneness to rupture due to a thin and highly inflamed fibrous cap. Concomitantly, we observed a high expression of MMP-9, indicating cap collagen degradation [22]. Interestingly, a significant increase of MMP-9 expression was also quantified in less progressed plaques from mice irradiated acutely with 0.3 Gy, indicating that MMP-9 secretion by macrophages is influenced by irradiation, in agreement with *in vitro* [23] and *in vivo* [24] studies underscoring a key role of radiation in modulating balance between MMP-9 and tissue inhibitor of metalloproteinase-1 (TIMP-1) levels.

Finally, although the pattern of radiation-induced aortic alterations and their severity increased at 6 Gy compared with a 20-fold lower dose of 0.3 Gy, our results tend to be far from linearity, and suggest that lower doses, such as those typically received in the nuclear workplace or from diagnostic examinations such as CT scanning, may be more damaging than predicted by a linear dose response. Although these are initial studies, they are in agreement with previous results from Mitchel *et al*. about nonlinearity of effects [18] and support ICRP recommendations [3] suggesting that clinical practice and workplace dose limits may have to be amended to afford adequate protection. In contrast with results from the same study [18], no protective effects of short-term exposure to a moderate dose of 0.3 Gy were observed in our experimental conditions.

## MATERIALS AND METHODS

### Animals

*ApoE* knockout (*ApoE^−/−^*) mice on C57BL/6J background (Charles River Laboratories, Calco, Italy) were bred at the animal facility of ENEA, under conventional conditions, or at the IES animal facility under specific pathogen free (SPF) conditions.

All experiments were conducted according to the Directive 2010/63/EU of the European Parliament on the protection of animals used for scientific purposes and to the legal regulations in Japan, respectively. Institutional Animal Care and Use Committees of both ENEA and IES approved this study. In both animal facilities, mice were fed a standard rodent chow *ad libitum* and maintained at 12 h light/dark cycle.

### Irradiation

At 8 weeks of age, female mice were randomly divided into 6 groups (*n* = 18∼24 each) and subjected to acute or chronic irradiation. Controls were sham-irradiated. For each group, 8 animals were used for morphometric analysis of heart and aorta, while tissues from 10–16 mice were used for molecular analyses whose results will be the object of a separate study.

Acute irradiation was performed at ENEA with single doses of 0.3 or 6 Gy x rays as described [25], dose rate: 0.89 Gy/min. For low dose rate exposures, mice were chronically irradiated at the IES with gamma rays from a ^137^Cs source (22 hr/day) reaching cumulative total doses of 0.3 or 6 Gy in 300 days (dose rate of 1 mGy/day and 20 mGy/day, respectively).

For acute irradiations (0.3 and 6 Gy) mice were euthanized by CO2 asphyxiation 300 days after exposure. For chronic irradiation, mice were analogously euthanized at the same age (360 days), after reaching cumulative doses of 0.3 or 6 Gy at the established dose rates. Hearts and thoracic aortas were collected from irradiated and sham-irradiated mice.

### Plaque morphometry

Aortas (*n* = 8) were processed as described [26], cut longitudinally, pinned *en face* on wax foils and subjected to Oil-Red-O (ORO) stain. Images were captured with a Leica digital camera, and different parameters were analyzed (i.e., percentage of plaque covered area over total area; number of plaques per unit area; plaque number and area) in the region from the aortic arch down to the diaphragm by image analysis software LASCore (Leica Microsystems, Milan, Italy).

### Histology and immunohistochemistry

Hearts and thoracic aortas (*n* = 6 each) were fixed in 10% neutral buffered formalin, and paraffin embedded according to standard protocols. Heart sections (4 μm) were cut in a plane perpendicular to the aorta axis. When the aortic root was identified by appearance of aortic valve leaflets, serial sections were collected and stained with H&E, Masson's trichrome or subjected to immunohistochemical and morphometric analyses [NIS-Elements BR4.00.05 (Nikon Instruments S.p.A., Firenze, Italy) and HistoQuest (TissueGnostics, Vienna, Austria)].

Antibodies: CD68 (polyclonal, AbCam, Cambridge, UK; 1:1000); α-SMA (monoclonal, Sigma-Aldrich, St. Louis, MO; 1:400); MMP-9 (polyclonal, AbCam; 1:50).

### Statistics

Data shown are means ± SEM; *t* test was used for determination of statistical difference between groups. Analyses were performed using GraphPad Prism 5.0 for Windows (GraphPad Software, San Diego, CA). *P* < 0.05 was considered statistically significant.
